# New genomic techniques and their European Union reform. Potential policy changes and their implications

**DOI:** 10.3389/fbioe.2022.1019081

**Published:** 2022-09-30

**Authors:** Tomasz Zimny

**Affiliations:** Institute of Law Studies, Polish Academy of Sciences, Warsaw, Poland

**Keywords:** GMO, new genomic techniques, biosafety, authorization, committee procedure, bioeconomy, precautionary principle

## Abstract

The article discusses amendment options (no significant change, lowering of administrative burdens or exemption of certain products from the legislation) for the European Union (EU) authorization procedures of New Genomic Techniques’ (NGT) products and their consequences for the sector and research institutions, particularly in the context of internal functioning, placing products on the market and international trade. A reform of the EU regulatory system requires a change in the procedures for the authorization of NGT products, otherwise EU researchers and investors may still be at a competitive disadvantage (as compared to Argentina, Brazil, Canada, United States or the United Kingdom) due to the inefficiency of the current system and the committee procedure for authorization. New legislation, currently being adopted in the United Kingdom is also presented for comparison.

## 1 Introduction

A process of revising the GMO legislation is currently ongoing in the European Union (EU). After preparing a study and two rounds of consultation, the European Commission (EC) plans to have a project ready in the second quarter of 2023. In the study (European Commission, 2021), the Commission mentioned that the current legislation may not be adequate to regulate research and marketing involving some products of “New Genomic Techniques” (NGTs) and indicated a need to alter it. In the new legislation the restrictions on research and use of regulated products are supposed be proportional to the risks connected with their use. The amendments also should contribute to the achievement of the goals of EU Green Deal and Farm to Fork strategies, which would require a more widespread use of such products, a higher throughput in authorization, and a higher level of legal certainty as to the outcomes of an authorization process. The term NGTs, is “an umbrella term to describe a variety of techniques that can alter the genetic material of an organism and that have emerged or have been developed since 2001, when the existing GMO legislation was adopted” (European Commission, 2021, 62). The glossary explains that it means at least: gene editing techniques either through the application of oligonucleotide mediated mutagenesis (ODM), site directed nucleases (SDN) (Zimny and Sowa, 2021) and RNA-directed DNA methylation (European Commission, 2021, 62–63), although this classification of methods and their products is not uncontroversial (Vives-Vallés and Collonnier, 2020; Van Der Meer et al., 2021).

According to the current EU GMO legislation any GM product requires authorization as food or feed (Regulation 1829/2003/EC, 2003) or another type of product [e.g., for cultivation (Directive 2001/18/EC, 2001)]. Such products need to undergo rigorous risk assessment (see e.g. Regulation 503/2013, 2013), and need to meet traceability and labelling criteria afterwards. Member states have significant flexibility in opting out from authorization of products meant for cultivation in the EU (Directive 2015/412/EU, 2015). Both the study and the amendment initiative are a result of a judgement of the Court of Justice of the EU, according to which only products of methods of mutagenesis routinely used until 2001 are exempted from the EU GMO legislation (CJEU C-528/16, 2018). Since the passing of this judgment multiple stakeholders proposed changes to the EU legislation, usually through exclusions or exemptions of certain classes of organisms, (e.g., featuring single nucleotide variants products of SDN 1 or 2 techniques or cisgenesis), (Zimny and Eriksson, 2020).

A thorough critique of the current EU regulatory system was performed by Eriksson and others in 2020 (Eriksson et al., 2020c; 2020b; 2020a). The authors indicated problems ranging from the current legislation’s unclear scope and conditions for authorization of products, through risk assessment procedures and their fitness for the purpose of performing proper risk management with regard to regulated products, and also problems with the post-authorization functioning of the products on the market. Proposed solutions to the problems involved: reconsideration of the current labelling requirements for authorized products, amendment of rules for the certification of organic products (Eriksson et al., 2020a), adding flexibility to the risk assessment procedures (to make the required steps dependable on the features of the examined product), switching from maximum to minimum harmonization in risk management (Eriksson et al., 2020b) and changing the approach to the regulation of organisms to a more product-oriented one, coupled with institutional and legal changes aimed at an increase of the certainty of law with regard to the development and marketing of regulated products (a pre-approval system) (Eriksson et al., 2020c). Issues, connected with the feasibility of the current legislation for the regulation of certain NGT products (in particular connected with detection and traceability), were risen by other authors (Emons et al., 2018; Broll et al., 2019; Sowa et al., 2021). Others postulate that risk assessment requirements should be altered with respect to products of targeted mutagenesis featuring small changes in the genome (Naegeli et al., 2020; Garcia-Alonso et al., 2022). The current authorization procedures also take ca. 5–6 years to complete (Smyth et al., 2014; Garcia-Alonso et al., 2022) and are rather costly [over 11 million € (Garcia-Alonso et al., 2022)], creating a high entry threshold for potential developers.

Criticisms of the current authorization system of GMOs also include the fact that the draft decisions by the EC may be accepted or rejected by a political body – a committee. The decisions regarding authorization of GM products in the EU are taken in “the committee procedure”, where a draft decision of the EC is submitted for deliberation to a committee comprising representatives of the member states of the EU. In case of GMOs it is the Genetically Modified Food and Feed and Environmental Risk section of the Standing Committee on Plants, Animals, Food and Feed (PAFF). The committee can either accept or reject the Commission’s decision or adopt no opinion. Acceptance and rejection require a qualified majority, hence if it is not reached, the Committee does not pass an opinion. In such a case or in the case of rejection, the draft is submitted to the Appeal Committee, operating on the same principles. If at this stage the opinion is favourable or no opinion is passed, the Commission can adopt the draft decision (see further Zimny et al., 2019). The role of the committee procedure is to involve member states in the decision-making process, when the EC issues a delegated or implementing act (e.g., a decision). The procedure is regulated by articles 290 and 291 of the Treaty on the Functioning of the European Union and the Regulation 182/2011 of the European Parliament and the Council (European Parliament and the Council, 2011). Currently there are multiple committees operating under the auspices of different directorates of the EC, and these committees are divided in to thematic subsections. This structure would suggest that the opinions of a committee have a form of a quasi-expert opinion, since particular subsections make decisions in a particular area of regulation. This does not seem to be the case, however, in the area of authorisation of genetically modified organisms.

**FIGURE 1:**
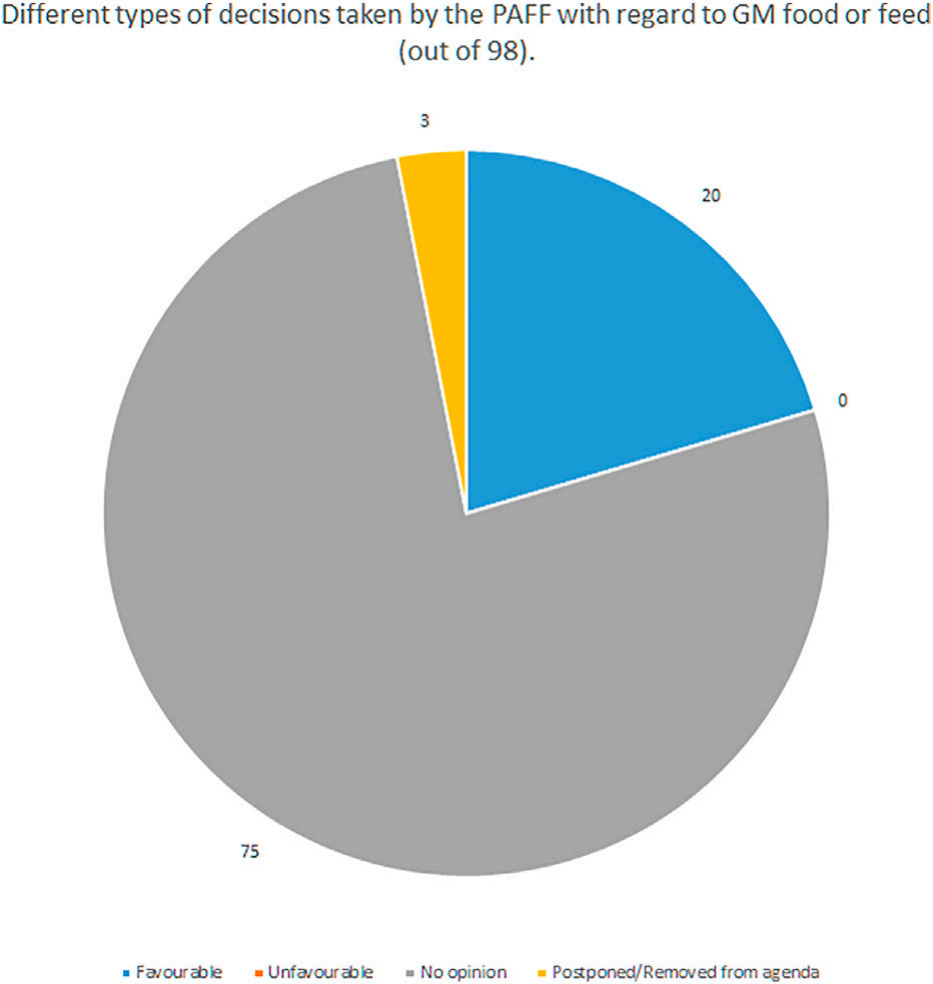
Different types of decisions taken by the PAFF with regard to GM food or feed between 10.2014 and 1.2022 (Zimny, 2022).

Committee members rather act upon the directives from their respective governments than basing on the scientific data. The committee can adopt or reject a decision with a qualified majority (55% of member states, no less than 15, comprising min. 65% of population). Notably this same majority is required for the adoption of changes to the GMO release directive, and some scholars indicate that reaching it after Brexit may be difficult due to the fact that the United Kingdom usually was generally in favour of transgenic crops (Purnhagen and Wesseler, 2021, 1631). An analysis of decisions taken by the PAFF between October 2014 and January 2022 shows that out of 98 decisions taken, 75 failed to reach the qualified majority, hence resulted in no opinion. Out of the remaining 23, 20 contained a favourable opinion, there were no unfavourable opinions (see Figure 1). All the favourable opinions were passed on purely formal issues, like the changing of the data of the applicant’s representative (Zimny, 2022).

The EU legislation on GMOs is based on the precautionary principle (PP), which obliges decision makers to undertake preventive measures in situations, where the knowledge about the undesired outcomes of a planned action (e.g., introduction of a new product to the market) is insufficient. The principle defines the EU’s approach to the protection of human health and the environment and is mentioned in art. 191.2 of the Treaty on the Functioning of the European Union. The CJEU mentioned PP as one of the reasons for its decision in the C-528/16 case (par. 50, 52, 53, 83), yet without going into a detailed analysis of its applicability to particular methods of gene editing, rather deciding about the products of such methods *en masse*. It should be noted that not every situation of uncertainty justifies the application of the PP. Its application should be necessary in the context of the lack of knowledge about the consequences of a given action (Zetterberg and Edvardsson Björnberg, 2017, 36). Where risks are known, preventive rather than precautionary measures, tailored to those risks, should be applied (Bergkamp and Hanekamp, 2018, 219). Excessive regulation (unjustified in view of possessed scientific knowledge) of an area of human activity may be viewed as a violation of the principle of proportionality (mandating that restrictions of basic freedoms should be genuinely necessary and justified), mentioned in art. 5.1 of the Treaty on European Union and art. 52.1 of the EU Charter of Fundamental Rights. For instance, if two groups of products are comparable in terms of risks connected with their use, and one group of such products is lightly regulated (e.g., products of conventional breeding or random mutagenesis), while another group is heavily regulated, then the regulation of the latter group may be seen as a violation of some basic freedoms (e.g., to conduct a business or freedom of arts and sciences). When drafting the new provisions on the authorisation of various NGT products, the EC needs to consider the PP on the one hand and the principle of proportionality on the other. The application of PP to certain products may no longer be justified, or certain regulations supposedly based on it, may no longer be necessary. This is a position taken *inter alia by* some of the EU’s major trade partners genetically modified goods, who have decided to lessen the regulatory burdens placed on plants not featuring stable insertions of foreign DNA fragments (Dederer and Hamburger, 2019).

Recently, the United Kingdom seems to have reacted to the criticisms of the current GMO legislation, by changing its laws with regard to certain NGT products. The amendment to the regulation on the deliberate release of genetically modified organisms (UK Parliament, 2022a) allows for an exemption from the risk assessment before experimental release of a “qualifying higher plant” (*inter alia* SDN 1 or 2 products, plants with epigenetic changes or certain cisgenesis products (ACRE, 2022). The second stage of the reform planned in the United Kingdom encompasses changes regarding obtaining, importing and marketing products of “precision breeding”. A bill proposing changes to the existing legislation was read in the House of Commons on the 25^th^ of May 2022 (UK Parliament, 2022b). The act (which shall apply to plants and animals–subject to welfare assessment) introduces a concept of a “precision bred organism”. Marketing of such organisms, will only be allowed, when such an organism would be a “marketable precision bred organism” (Art. 5 (1a)) or its “qualifying progeny” (Art. 5 (1b)) and 24), subject to a confirmation issued by the Secretary of State upon receipt of a report of an advisory committee (issued within 90 days). Marketing of food and feed products shall to a large extent be subject to regulations, which may impose obligations regarding obtaining a marketing authorisation and impose traceability requirements (Part 3 of the bill). It is yet too early to predict if the bill will be passed in the form it was submitted to the House of Commons, but its tenor indicates that the United Kingdom wishes to follow in the footsteps of other EU’s important trading partners, who severely lessened the regulatory burden placed on NGT products, which would otherwise be obtainable through conventional breeding or random mutagenesis, or do not feature stable inserts of foreign DNA fragments—e.g., Argentina, Brazil, Canada, the United States (Dederer and Hamburger, 2019; USDA, 2020; Zimny and Sowa, 2021). Lack of harmonization of regulations with such countries might result in serious cost increase and regulatory burdens placed both on the EU authorities and entrepreneurs (Ryan and Smyth, 2012).

## 2 Policy options and implications

The outcome of the Commission’s initiative to amend the legislation is currently uncertain. The questionnaire for the recently concluded poll contained a whole spectrum of options, from a lack of changes, to changes envisioning a departure from risk assessment requirements for certain products. The project may not be adopted by the EU before the Commission’s term of office runs out in October 2024. Given that the Commission has declared a need for a legislation, in which the regulatory burdens would be proportional to the risks connected with the use of the product in question, it needs to prepare a project that would comply with both the PP and the principle of proportionality. Taking this into consideration one can distinguish three policy options: 1—no change or negligible changes to the legislation, 2—limited changes, in particular through restrictions in the risk assessment requirements, 3—exemption of certain products from the legislation, in particular products featuring changes that would also be achievable through conventional breeding or random mutagenesis.

### 2.1 No changes or negligible changes

This option essentially means the maintenance of the *status quo*, which is not a scenario desired by the EC or the stakeholders advocating a reform of the legislation. According to the current interpretation of the definition of the GMO, products of modern methods of gene editing will fall under the current GMO legislation with all its drawbacks (see above). This scenario would be marked with a low throughput of the authorization procedures (Smyth et al., 2014; Garcia-Alonso et al., 2022), uncertainty of their outcomes strengthened by the politicization of the decision-making process (Purnhagen, 2019), problems with international trade of such goods (Purnhagen and Wesseler, 2021; Zimny and Sowa, 2021) as well as increased costs of authorization, but also potential occurrence of unauthorized products imported from third countries (Ryan and Smyth, 2012; Purnhagen and Wesseler, 2021). These consequences would significantly limit the economic justifiability of choosing an NGT for the development of products for the EU agricultural market. The application of the current GMO regulatory framework to some of the NGT products (an inevitable consequence of a lack of changes in the regulatory approach) can be seen as overregulation, not justified by the PP nor the proportionality principle (see below). The mere fact that a certain requirement can technically be introduced, does not make such a requirement scientifically or legally justified.

### 2.2 Limited changes

The actual contents of the EC project are not known yet, however the questionnaire for the survey, which ended in July 2022 contained a wide variety of options, including, *inter alia*:

- adapting risk assessment requirements for plants produced through targeted mutagenesis or cisgenesis (Question 3);
- introduction of a fast track authorization system or fee reductions for plants with traits contributing to sustainability (Question 7);
- waiving or limiting the duty to develop a method for detection and differentiation of plants produced by cisgenesis or targeted mutagenesis, where such a method cannot be provided (Question 11);

and others (European Commission, 2022). The practicality of such solutions and their actual content is still subject to speculation, (e.g., the meaning of “traits contributing to sustainability”). In view of the European Food Safety Authority’s (EFSA) opinions regarding the applicability of the current GMO risk assessment requirements to products developed through SDN 1-3, ODM or cisgenesis, even if they are sufficient for the assessment, parts of those requirements may not be applicable or necessary for the determination of the safety of such products. Particularly, the assessment of SDN 1-2 and ODM products from the point of view of the safety of gene products could depend on the allele that was edited. Should the allele and the trait associated with it be already present in a cultivated variety, the risk assessment could be focussed on the history of safe use of said variety rather than on the specific data on the edited gene. This would not be the case for a completely new allele and trait (Naegeli et al., 2020, 8). Similarly, it is expected that the number of off target mutations for such products may be comparable with those of conventional breeding methods, and the existing environmental risk assessment requirements, while sufficient for the evaluation of SDN 1-2 and ODM products, would only partially be applicable to them, due to them featuring a modification of an endogenous sequence rather than an insertion of a transgene (Naegeli et al., 2020, 10).

While resignation from some risk assessment elements, justified by the lack of a stably present insert would not be appropriate for cisgenic products, there is still a leeway when it comes to such products, on a case by case basis, particularly if the familiarity of the plant and introduced gene were to be taken into consideration. Requirements justified by risks connected with the introduction of a foreign gene could be to an extent limited for such products as well. EFSA deemed parts of the abovementioned requirements currently applied for “classic” GMOs not applicable to cisgenic products (EFSA Panel on GMO, 2012, 18–19).

Changes in the authorisation procedures in this scenario could then encompass at least (EFSA Panel on GMO, 2012, 19; Naegeli et al., 2020, 8, 11):

- lower requirements for experimental data for SDN 1-2 and ODM products (lack of transgene or cisgene);
- lower data requirements on the safety of gene products SDN 1-2 and ODM products basing on the familiarity of the altered alleles and traits;
- no risk assessment of the transgene itself (due to the lack of it);
- on a case by case basis: lower data requirements for cisgenic products, basing on their familiarity;
- and additionally a system that would facilitate the authorisation of the abovementioned products, at least through an *ex ante* status confirmation.

The adoption of such solutions (reduction of risk assessment requirements, a “fast track” for certain known products) would definitely lessen the administrative burdens placed upon researchers and developers of such products. Among the benefits, from their point of view, one can mention an increased throughput of the authorization process, lower uncertainty of as to the outcomes of that process, lowering of the costs of performing the risk assessment and obtaining the authorization, particularly if a pre-approval system for some products would be introduced. However, the ultimate decision would still depend on the political vote within a committee. If labelling requirements for GMOs would be maintained also for products of targeted mutagenesis or cisgenesis, this would still hamper the international trade with countries not having such requirements (see above) and result in potential stigmatization of labelled products, their removal from production chains, hence cause a lowered demand for such products.

### 2.3 Exemption of certain products from the legislation

Exemption (the way products of random mutagenesis are currently exempted) of certain products from legislation (e.g., SDN-1 and 2 - Vives-Vallés and Collonnier, 2020; see also Zimny and Eriksson, 2020) or even an interpretation of the GMO definition in such a way that it would not cover such products (Van Der Meer et al., 2021) has been postulated not only by researchers, but also stakeholders and some organizations. The adoption of such a policy would definitely have the most benefits from the point of view of researchers and developers of products covered by the exemption. An exempted product does not need to undergo any authorization procedures, which are also not required for non-regulated products [e.g., variety evaluation for the purposes of its placing in the Common Catalogue–an EU database of registered plant varieties, which are no longer subject to marketing restrictions (European Parliament and the Council, 2002)]. Access to the market of such products and the costs of their marketing would be greatly improved. Also the legal certainty of investors would be significantly enhanced, since the access to the market would no longer depend on a decision of a political body. Such a solution would also be harmonized with the systems adopted by the aforementioned trade partners, including the new legislation currently discussed in the United Kingdom. Introduction of a pre-approval system that would determine the legal status of a product before its development, as has been postulated in the literature, (Eriksson et al., 2020c), would further facilitate the decision making process on the side of the researchers and investors.

It needs to be stressed that with sufficient information available, an exemption of some products from the regulation does not need to result in a violation of the PP. If the risks connected with the use of a certain plants for their intended purpose were to be sufficiently known, and if they were deemed to be comparable with those associated with the use of already exempted plants, then preventive measures, such as a status confirmation system or supervision at the development level, could be sufficient to satisfy the safety requirements. Particularly if this solution were to be applied to products of SDN 1-2, ODM edition of known alleles with a history of safe use. The prerequisites for such an exemption should be cautiously determined by an expert body (e.g., EFSA GMO Panel), taking multiple factors into consideration, and be subject to periodical review.

The adoption of this policy option, even for a limited group of plants, would however have some significant drawbacks. Firstly the official control over such products would be much lower than in the remaining scenarios discussed here. Transparency, particularly perceived by the general population would also suffer, with lack of official oversight and reporting or labelling duties. This might lower the trust in the biosafety system as such. These features may render this policy option the least likely to be adopted, since it may be difficult to find political support for it (Purnhagen and Wesseler, 2021, 1631–1633). Another potential drawback may be the fact that as per the CJEU judgment, the member states of the EU are able to introduce national restrictions on exempted products. This policy option would be the easiest to implement, due to the lack of administrative burdens and special regulatory provisions connected with them.

## 3 Actionable recommendations

There are actually two stages of actions to be taken, depending on the state of adoption of the prospective amendment of the EU legislation regarding NGT products. The first stage –before the adoption of a project and with the preliminary consultations already closed, would involve participation in public activities for the stakeholders, aimed at the preparation of a project that would ease the administrative burdens, as well as harmonize the legislation with that of EU’s closest trade partners and neighbours.

Should the EU succeed in adopting a new legislation that will comprise at least the solutions presented in options 2 or 3, the formal situation of researchers will become more complicated than currently. Instead of having to consider three categories of organisms, as is currently the case [non-GMOs, regulated GMOs and GMOs exempted from legislation (Custers, 2017)] they may need to consider several additional categories–various NGT products that will legally be GMOs with an altered level of regulation. The legal status of a given organism will heavily influence its future viability as a product, depending on the requirements for research and marketing placed on it. Given that many R&D units will still employ a variety of methods in their activities, two types of solutions may help with the inter-institutional decision making process, as regards the choice of breeding methods and compliance. Firstly the development of an internal policy document, or even an algorithm that would help researchers with determining the legal status of their products depending on the methods and nature of intervention into the plants’ genome. Secondly, the decision making process may be supported by establishment of an advisory body comprising compliance officers or persons otherwise competent in the assessment of the regulatory status of certain products, whose opinion would facilitate the decision-making process within the institution.

## 4 Conclusion

Despite the declarations of the EC regarding the amendment of the legislation, the future of NGT products in the EU still remains uncertain. Even if changes lessening the regulatory burdens placed on plants resulting from the use of NGTs are going to come into force, it is not clear that they will satisfy the needs of the R&D sector. The United Kingdom seems to follow into the footsteps of other EU’s trade partners in agricultural goods, through a significant lessening of regulation of products, which could otherwise be obtained through methods of conventional breeding or random mutagenesis. Adoption of any amendments in the EU will require a proper response and policy adjustment on the part of the research institutions as well.
